# Dietary habits, physical activity, and sedentary behaviour of children of employed mothers: A systematic review

**DOI:** 10.1016/j.pmedr.2021.101607

**Published:** 2021-10-22

**Authors:** Sabiha Afrin, Amy B. Mullens, Sayan Chakrabarty, Lupa Bhowmik, Stuart J.H. Biddle

**Affiliations:** a University of Southern Queensland, Centre for Health Research, Springfield, Australia; b Shahjalal University of Science & Technology, Sylhet, Bangladesh; c University of Southern Queensland, School of Psychology and Counselling, Ipswich, Australia

**Keywords:** Maternal employment, Children and adolescent, Dietary patterns (DP), Physical activity (PA), Sedentary behaviour (SB)

## Abstract

Since approximately 40% of the global workforce are women, a comprehensive understanding of association of maternal employment with child dietary patterns, physical activity and sedentary behaviour needs more focus. This systematic review aims to identify the association between maternal employment and dietary patterns (DP), physical activity (PA) and sedentary behaviour (SB) of children and adolescents (6 to 18 years). Searches were performed using electronic databases and manual searches. Peer reviewed journal articles, conference papers, theses at masters/doctoral levels in English were included. A total 42 studies met selection criteria, which indicated associations between maternal employment and at least one of the domains of interest: DP, PA and/or SB. Using individual samples of analysis, it was found that, 9 samples of DP, 11 samples of PA and 12 samples of SB were positively correlated with maternal employment, whereas 25 samples of DP, 5 samples of PA and 5 samples of SB showed an opposite association. Results suggest that PA and SB were positively related with maternal employment, whereas DP had an inverse relationship. Findings from this review provide evidence that children of employed mothers had poorer DP and greater prevalence of SB, however, their children are more physically active. Future interventions need to create a positive environment at the workplace and for families to support employed mothers and improve children’s dietary patterns and decrease sedentary behaviours. Future studies should prioritise the domains of DP, PA and SB that have been studied inadequately and have inconsistent results.

## Highlights

• Dietary pattern is poorer among children of employed mothers.
• Children of employed mothers are more physically active.
• Children of employed mothers experience greater prevalence of sedentary activity.

## 1 Introduction

Two important worldwide trends can be identified in recent years: increasing prevalence of childhood overweight/obesity and increasing participation of women in the paid labour force. Childhood obesity is an emerging salient public health challenge of the 21st century (WHO, 2020). Childhood obesity is risky as it has strong associations with likelihood of adult obesity, which has led to the increasing risk of morbidity, including non-communicable diseases (NDCs) such as cardiovascular disease, type 2 diabetes mellitus, some cancers, poor skeletal health, and some aspects of mental health (Biddle et al., 2004, Das and Horton, 2012, Lee et al., 2012, Wolin et al., 2010).

According to World Development Indicator (World Bank, 2020), worldwide female employment rate increased substantially in the last century. Employment creates a double burden for women as they often take the family responsibilities of unpaid household tasks and childcare due to traditional division of labour. Balancing with daily multiple roles and responsibilities, employment may impact upon the wellbeing of children if, as hypothesized, employed mothers spend less time on household activities centred on children, such as children’s diet and physical activity (Bianchi, 2000, Cawley and Liu, 2012). However, employment may contribute to greater economic opportunities and resources, which may also enhance health and wellbeing (Waddell and Burton, 2006).

Literature from the USA (Datar et al., 2014, Anderson et al., 2003), U.K. (Hawkins et al., 2008), Canada (Chia, 2008) and Germany (Baten and Böhm, 2010) have demonstrated that children of employed mothers demonstrate a trend towards being overweight due to changes in food intake patterns (e.g., homemade food vs meals from outside, more processed food and ‘junk’ food), reduced physical activity and increased sedentary behaviour. The latter is defined as sitting or lying with low energy expenditure during waking hours (Tremblay et al., 2017). Poorer health behaviours among children (e.g., unhealthy dietary patterns, physical inactivity, sedentary behaviours) serve as gateways towards poorer health trajectories and increased health comorbidities in adulthood, including being overweight and obese (Mu et al., 2017). Childhood adiposity as well as physical inactivity and sedentary behaviour among children; represent key modifiable risk factors, to enhance both current and future health outcomes (Raynor et al., 2012).

There has been a visible shift in women’s employment in low to middle-income countries (LMIC) over the past two decades (Dodzin and Vamvakidis, 2004, Lopez-Arana et al., 2013). The increase in women’s participation in the labour force parallels the increasing prevalence of overweight among children (BMI Z-score > 2), which is believed to occur as a result of the country’s nutrition transitions (referred to as characteristic changes in food and physical activity patterns that occur as a result of macro-level changes in economic development, globalization and urbanization) (Lopez-Arana et al., 2013). While the literature confirms that children of working mothers in developed countries demonstrate a trend for being overweight (Datar et al., 2014, Anderson et al., 2003, Baten and Böhm, 2010), research related to dietary patterns, physical activity and sedentary behaviour of children in LMICs are scarce to confirm any definite relationship. Thus, the relationships between maternal employment and children’s dietary patterns, physical activity and sedentary behaviour are largely unexplored in LMIC.

No systematic review has investigated how dietary patterns, physical activity and sedentary behaviours (in combination) among children are related to maternal employment. Some previous research that has been identified investigated the association of maternal employment with child obesity and discussed solely one or two of these behaviours as influencing factors towards overweight and obesity (Duch et al., 2013, Mech et al., 2016, Hoyos Cillero and Jago, 2010, Shrewsbury and Wardle, 2008). However, not only single behaviours, but the combination of multiple risk behaviours ultimately determines the risk of being overweight or obese. It is already identified that consumption of energy dense foods, low levels of physical activity and high levels of recreational screen use (e.g., TV watching and computer use) are key behavioural determinants of overweight and obesity in children and adolescents (Barnett et al., 2018, Rennie et al., 2005). Dietary and physical activity habits are developed at early stages of life (Savage et al., 2007) and have been tracked into adulthood (Kelder et al., 1994, Tammelin et al., 2014), suggesting the importance of increasing our understanding of the roots and development of these behaviours in children. The relationships among maternal employment and these three key behavioural variables remains largely unknown. Since approximately 40% of the global workforce are women (World Bank, 2020), a comprehensive understanding of association of maternal employment with child dietary patterns, physical activity and sedentary behaviour needs more focus considering its short- and long-term impacts on health and wellbeing trajectories over the life course.

This systematic review aims to identify the association between the dietary habits, physical activity and sedentary behaviour of children, with the employment status of mothers. Our main research question is, therefore, ‘does the employment status of mothers with children aged 6–18 years affect children’s dietary habits, physical activity and sedentary behaviour?’

## 2 Methods

The research protocol of this study is registered in PROSPERO, an international prospective register of systematic reviews (registration number: CRD42020145438).

### 2.1 Search strategy

The review followed the protocol of the Preferred Reporting Items for Systematic Reviews and Meta-Analyses (*PRISMA*) guidelines (Moher et al., 2009). Literature searches were conducted using the bibliographic databases of Scopus, PubMed, Science Direct, JSTOR, Google Scholar and ProQuest. For the primary search, no restriction was imposed on publication type, or study design, however, only English language papers were considered. Initial search was applied to title, abstract and key words. For working mothers and physical activity, the following search terms were used: (physical activity OR physical inactivity OR exercise OR sedentary behaviour) AND (maternal work OR working mother OR maternal employ* OR working women) AND ('child' OR 'adolescent' OR 'youth' OR 'juvenile'). For diet, ‘dietary pattern OR dietary intake OR food intake OR dietary habits OR feeding behaviour’ were used instead of 'physical activity' or 'sedentary behaviour'. Additional literature and document searches were undertaken via backward searching through the key words identified from the literature review and the secondary literature search of the reference lists of all full text articles selected in the primary search. The search strategies are presented in Supplementary Material.

### 2.2 Inclusion criteria

Studies were incorporated in the present review if they (i) reported on maternal employment status, and dietary patterns (DP), physical activity (PA), or sedentary behaviour (SB) of children aged 6 to 18 years; (ii) were written in English; (iii) published as a peer reviewed journal article, conference paper, or thesis at masters or doctoral level. All research methods, designs, as well as measurement instruments were included. Studies were not considered for inclusion in the systematic review if: (i) the population of the study was only adults, (ii) had obesity or overweight as the focal point of research outcomes, (iii) published as a literature review, (iv) did not provide information about the age of the study population, (v) full text was not available and (iii) was not written in English.

### 2.3 Study selection and data extraction

All studies identified by database search and additional searches were screened for eligibility based on title, abstract and full text by two independent reviewers (SA and LB). Any disagreements were resolved by discussion with the other reviewers/authors.

The data for each included study were extracted by the first author using a standardized extraction form and verified by other authors. Data were extracted on: (1) characteristic of publication [title of the article, author(s), year, country/data source, study design], (2) sample characteristics [sample size, age/age group, employment status/working hours], (3) primary and secondary outcomes as well as measurement methods used for dietary patterns, physical activity and sedentary behaviour. A narrative synthesis of results of included studies was provided. The number of studies included in the systematic literature review were too diverse in outcome as well as measurement to pool data to conduct a *meta*-analysis.

### 2.4 Coding associations with dietary outcome, physical activity and sedentary behaviour

Studies with significant associations identified between maternal employment and domains of dietary patterns, physical activity and sedentary behaviour were not discussed unless three or more studies were available (for each category) (Sallis et al., 2000). Conceptually similar domains were combined if there were not enough studies to examine the domains individually. For example, ‘snack food including fast food & junk food’ domain combined fast food, junk food and processed food. Domains relating to physical activity in included studies were too diverse to report on separately, thus conceptually similar domains were aggregated as moderate to vigorous intensity physical activity (MVPA).

Studies with significant associations between maternal employment and variables (dietary patterns, physical activity and sedentary behaviour) were included in the ‘Related to maternal employment’ column of Table 2; and associations were classified and coded as: positive association (+), negative association (−). Studies reporting no significant associations were entered in the “Unrelated to maternal employment” column. The coding process was completed following the rules used by Sallis et al. (2000). Studies with low risk of bias scores are presented in bold numbers in the Table 2. Included studies typically used univariate tests for assessing the statistical significance of associations. However, even if multivariate tests were conducted, univariate tests were reported for consistency across studies to ensure meaningful comparisons of key findings.

### 2.5 Summary codes

Numbers in the second and the fourth columns of Table 2 refer to the study numbers in Table 1. Studies that examined multiple domains of dietary patterns, physical activity or sedentary behaviour, multiple associations with maternal employment were recorded. The column ‘number of samples’ includes the number of samples that have been studied for each identified domain. The ‘Summary’ column contains a code to summarize the state of the domain for that variable. After assessing all the studies, calculating the percentages of findings supporting the overall association, each domain was classified as no association (0%–33 % of studies supporting the association), indeterminate/inconsistent (34%–59 % of studies supporting the association) and positive or negative association (60%–100 % of studies supporting the association) and coded as ‘0′, ‘?’ and ‘+/−’ respectively. These rules for classifying variables strength of evidence and direction of association are in accordance with Sallis et al. (2000).

**Table 1:** Characteristics of included studies, along with the results of the study quality assessment for each study (n = 42).

**Study**	**Type of country**	**Study design**	**Sample size, source of data, recruitment method**	**Empirical method / Theoretical framework**	**Measure of maternal employment**	**Measure of dietary patterns (DP)**	**Measure of physical activity (PA)**	**Measure of sedentary behaviour (SB)**	**Association with maternal employment**	**Risk of bias**
Brown et al., 2010^(1)^ Australia	HIC	Cross sectional	6–7 years (n = 4464) Child cohort of the second wave of the Longitudinal Study of Australian Children (LSAC)	Path model (multiple regression analysis)	Full-time employment= < 34 h per week, part-time = 1–34 h per week. Part-time employed 44%	Interview with 24 h diary of consumption	Interview with 24 h dairy (Walk for travel or fun’, ‘ride bike, trike etc for travel or fun’, ‘other exercise (e.g., swim, dance, run about)	Interview with 24 h dairy on watching TV, video, DVD or movie	Mother’s part time working status is negatively associated to television viewing and snack food consumption.	Low risk of bias
Vazquez-Nava et al., 2013^(2)^ Mexico	UMIC	Cross sectional study.	6–12 years (n = 897) Randomly selection	Logistic regression	Self-reported employment status. Employed mother = 38.8%.	Interview on dietary habits	Interview on sports practice.	Interview on play minutes per session / week and TV watching, video games.	Maternal employment had positive association with sedentary lifestyle	High risk of bias
Adbi et al., 2017^(3)^ India	LMIC	Cross-sectional study	13–17 years (n = 1416) Data collected from three public schools.	Simple chi-square test and multinomial logistic regression	Self-reported employment status	Self-administered GSHS questionnaire	–	–	Adolescents’ junk food intake was negatively related to working mothers	Low risk of bias
Sethi et al. (2014)^(4)^ India	LMIC	Observational study	7–9 years (n = 100) Data collected from two govt. schools.	Descriptive statistics	Self-reported employment status. Working mother = 70%	Interview with 24 h dietary recall method	–	–	Children food intake had negative association with working mothers	High risk of bias
Park et al. (2014)^(5)^ South Korea	HIC	Qualitative	10–16 years (n = 26) Data collected from 26 schools.	Thematic analysis (5 principal themes)	Self-reported employment status	In-depth interviews and focus group discussions	–	–	Eating out and minimal breakfast is positively related to maternal employment.	Low risk of bias
Neumark-Sztainer Det al. (2002) ^(6)^ USA	HIC	Cross-sectional	11–18 years (n = 4746) Data collected from 31 schools.	Cross tabulations, log-linear modelling, and linear regressions.	Self-reported employment status	Survey with YAQ	–	–	Maternal employment was negatively associated with family meal patterns.	High risk of bias
Honajee et al., 2012^(7)^ Mauritius	UMIC	Cross-sectional	2–11 years (n = 289)	Chi square test and factor analysis.	Self-reported as Professional worker	Self-reported FFQ	–	–	Healthier eating of children was significantly and positively associated to maternal employment.	Unclear risk of bias
Fitzsimons and Pongiglione, 2019^(8)^ UK	HIC	Longitudinal cohort study	9 months −14 years (n = 7, 894)	OLS and FE linear probability models	Self-reported employed if work in the last week or had a job and did not work in the past week for reasons other than parental leave. Part-time employment = 1 and 34 h, full-time = 35 h or more.	Interview about regularity of breakfast on every weekday	–	Interview on TV watching (exceeding three hours per weekday)	Children of employed (both part- and full time) mother watch more TV and less likely to have regular breakfast.	High risk of bias
Cho, 2017 South Korea ^(9)^	HIC	Cross sectional study	14.2 years (n = 1,873) Stratified multi-stage sampling technique.	OLS regression, logistic regression models and Sensitivity test	Mothers reported their daily start and end times at work. Average work hours per week calculated as [((weekday work hours × 5) + (Weekend work hours × 2))/7]	–	Interview on average hours per week in vigorous exercise	Watching TV/video/DVD and playing electronic (computer or video) game per day average time	Longer working hours of mothers was positively associated to TV/Video/DVD viewing, electronic game playing and inversely to physical activity.	Low risk of bias
Maher et al., 2017^(10)^ USA	HIC	Longitudinal study	8–12 years (n = 191) Students were recruited from school.	Linear regression model and Hochberg Procedure (to reduce Type I error)	Self-reported	–	Accelerometer measures MVPA	Accelerometer	Maternal employment status did not increase child physical activity or sedentary behaviour.	Low risk of bias
Chowhan and Stewart, 2014^(11)^ Canada	HIC	Cross-sectional study	12–17 years (n = 3591)	Linear probability model, FE and instrumental variable models.	Weeks employed in the previous year and the usual hours worked during each of those weeks	Interview on eating habits (eating breakfast every day) and allowance	Interview on Sports, gym class, dance, gymnastics, karate or other groups or lessons	Interview on average daily hours spend watching TV	More working hours of mother was positively related to TV viewing and negatively related to eating breakfast daily. More weeks worked by the mother was negatively related to TV viewing and positively related to physical activity.	High risk of bias
Ham et al., 2013^(12)^ South Korea	HIC	Cross sectional study.	10.01 years (n = 370) Data collected from students at elementary schools.	One-way analysis of variance tests, chi-square tests and multinomial logistic regression	Self-reported. Employed mother = 53.5%	Self-reported eating behaviour	Self-reported exercise	Self-reported screen time (time spent on TV/video/computer/video games)	Children of working mothers had significant positive association with screen time.	High risk of bias
Ziol-Guest et al., 2013^(13)^ USA	HIC	Longitudinal study	13 or 14 years (n = 4192)	OLS regressions and logistic regressions.	Weekly working hours	–	–	Interview on TV watching (Average number of hours)	More maternal working hours are positively associated with hours of TV watching.	Low risk of bias
Touliatos et al., 1984^(14)^ USA	HIC	Cross-sectional study.	10–13 years (n = 99) Data collected from school.	Factor analysis	Self-reported. Employed mother = 66%	Interviewed with 24 h recalls of dietary intake	–	–	Maternal employment and child dietary quality had positive association	High risk of bias
Gaina et al., 2009^(15)^ Japan	HIC	Cross-sectional study	12–13 years (n = 10453). Data collected from high school children.	*t*-test and χ2 analyses (or Fisher’s exact test), Binominal logistic regression	Self-reported. Full time employed mother = 50.7%; part time = 32.7%	Self-reported eating meals regularity, meals speed and amount	Self-reported physical activity measured in hours per week	Self-reported TV watching time and room tenure	Mothers’ employment has no effect on breakfast. Children of full-time employed mothers skip dinner. Children of part-time employed mothers snacked more, eat dinner regularly and eat larger meal portions compared with children of full-time employed mothers. Children non-employed mothers eat faster.	High risk of bias
Hsin and Felfe, 2014^(16)^ USA	HIC	Longitudinal study	0–12 years (n = 1127)	Individual FE and IV regressions	Average weekly hours worked. Employed mother = 78%	–	–	Self-reported time use survey on 24-hour periods (watching TV listening music, and unspecified leisure activities)	Full-time employed mothers had negative association with children’s unstructured activities (watching television, listening to music, and unspecified leisure activities)	Low risk of bias
Bauer et al., 2012^(17)^ USA	HIC	Cross sectional study	Adolescent recruited from middle and high schools. n = 2893.	linear regression models, generalized estimating equations	Working full-time; working part-time; stay-at-home caregiver; currently unemployed but actively seeking work; and not working for pay. Full-time employed mothers = 46%	Interview on family meal with questionnaire	–	–	Full-time employment of mothers had negative association with family meals, positive with fast food family meal and negative with fruit and vegetable intake.	Low risk of bias
Li et al., 2012^(18)^ Australia	HIC	Longitudinal (Prospective)study	1–14 years (n = 1629)	Multivariate linear regression models	Not working, working 1–15 h, 16–24 h, 25–34 h or ≥ 35 h (full time) weekly.	Self-reported Semi-quantitative FFQ for dietary intake		–	Increasing working hours of full time employed mothers were negatively associated with diet quality.	High risk of bias
Sweeting and West, 2005^(19)^ Scotland	HIC	Cross-sectional study	11 years (n = 2146) Data collected from school.	Logistic regression	Self-reported as full-time home maker, part-time work, full-time work and unemployed	Self-reported Questionnaires on healthy eating habits	–	–	The likelihood of less healthy eating is lower for the children of part-time mothers. Unhealthy snacking was not associated to maternal employment.	High risk of bias
Morrissey et al., 2011^(20)^ USA	HIC	Observational study.	3rd, 5th and 6th grade (N = 990)	RE and within-child FE regressions.	Self-reported	–	Physical activity monitor (seven consecutive days during a typical school week)	Interview on watching TV	Grade 3 children of employed mother watched an average of 15.2 h of television per week and spent about one-fifth of their time in moderate or vigorous physical activity. 5th and 6th grade children of employed mother had poorer food choices and more sedentary activity relative to younger age.	High risk of bias
Chia, 2008^(21)^ Canada	HIC	Cross sectional study	6–11 years (n = 4107).	OLS regression. Reduced form equation	Average number of hours of paid worked per week	–	Interview on organized and non-organized sports	Interview on watching TV and videos	Weekly hours worked by the mother was positively associated with probability of watching more hours of television or video programs per day. Mother’s weekly working hours is positive and statistically significant with regular participation in organized sporting activities.	High risk of bias
Chang and Lee, 2012^(22)^ South Korea	HIC	Cross sectional study	10 or older (n = 14228)	Two-part regression model of time (The first part regression used Probit and the second part was estimated OLS	Local unemployment rates as instrumental variables	Self-reported Two-day time diaries	Self- reported two-day time diaries on supervising or playing with children	–	Mothers employment had negative association with family meals, and supervision of children’s physical activity	Low risk of bias
Chang, 2012^(23)^ USA	HIC	Cross sectional study	12–17 years (n = 637) Stratified random sampling. Survey data	Multivariate multilevel linear regression	Average number of working hours per week.	Family dinner		TV watching	Children’s TV-watching hours were not associated with Primary Care Givers’ employment status	High risk of bias
Nadia (2012) ^(24)^ USA	HIC	Cross sectional study	Fifth grade (average age of children is about 10 years). Data (survey) collected from school.	Ordered Probit.	Number of hours worked per week. Full time employment= >20 h per week	Number of glasses of juice drink, number of times soda pop/sport drinks/fruit drinks drank, number of times green salad, carrots, other vegetables, fruits, and fast food eaten in last week.	Number of days per week of 20 min exercise.	TV watching	Maternal full-time employment was positively associated to watching TV and negatively to number of times child drank juice, ate carrots, other vegetables, fruits, and number of times child ate dinner regularly.	High risk of bias
Shuhaimi and Muniandy, 2012^(25)^ Malaysia	UMIC	Cross sectional survey	4–6 years (n = 142) Data collected from 7 Kindergarten.	Two-way ANNOVA, Pearson rank correlation.	Self-reported as unemployed and employed.	Self-reported children’s three-days food intake record	–	–	Negative association was found between maternal working hours and child’s energy, protein and fat intake; breakfast eating	Low risk of bias
Aniza I et al. (2009) ^(26)^ Malaysia	UMIC	Cross sectional study	14 and 16 years (n = 519) Data collected from secondary school students	Bivariate analysis and logistic regression	Self-reported employment status	–	Self-reported PA using International Physical Activity Questionnaire (IPAQ).	–	Maternal employment had positive association with physical activity	Low risk of bias
Martin et al., 2018^(27)^ USA	HIC	Longitudinal study	12–18 years (n = 10,518)	FE and cross-sectional model.	Hours a week does s/ he work for pay	–	Self-reported number of times they engaged in various leisure activities during the past week	Self-reported weekly hours of watching television, videos and playing video or computer games.	Positive association of screen time with mothers’ work hours and mothers’ unemployment.	High risk of bias
Meyer, 2016^(28)^ Germany	HIC	Cross-sectional study.	9–12 (n = 2447)	OLS, linear probability model, 2SLS and IV estimate.	Self-reported employment status	Self-report on combined measure of eating raw and cooked vegetables.	Self-reported frequency of physical activity (exercise) 3 times per week	Self-reported hours of watching TV/ playing video games per day.	Maternal full-time employment has positive relation to unhealthy dietary habits (lower consumption of fruits and vegetables, and a higher consumption of soda drinks and processed food) and watching TV, playing video games and activity behaviour.	High risk of bias
Nie and Sousa-Poza, 2014^(29)^ China	UMIC	Longitudinal study	3–17 years (n = 2618) Multistage random cluster sampling method	OLS and quantile regressions.	Hours worked during the past week	Self-reported dietary patterns: meals at home and caloric intake	Time spend on physical exercise (gymnastics, track, swimming and ball games) and other sports before or after school (measured in minutes per week).	Self-reported total time spent watching TV, doing homework, and reading and writing (measured in minutes per week).	Maternal working status is not significantly associated with caloric intake, meals at home, physical exercises, and/or sedentary activities.	High risk of bias
Anderson, 2012^(30)^ USA	HIC	Longitudinal study	Kindergarten to eighth grade.	Probit model	Working hours per week.	Regularity and frequency of eating meals at home, fast food, and eat snacks at school	Self-reported amount of time spent in physically activity (Days/week with vigorous exercise).	Family rules and actual amounts of television viewing (h/week)	Positive association of maternal employment on children’s organized activities and more working hours were negatively correlated with regular family meals, regular meal-times, and rules about television watching.	High risk of bias
Taylor et al., 2012^(31)^ Australia	HIC	Cross sectional study.	5–15 years (n = 614). Random sampling.	Univariate/multivariate logistic regression l	Full-time or part-time employment was determined according to a cut-off of 35 h per week.	Data collected via telephone monitoring system on fruit and vegetables, processed meat; fast food; potatoes; juice; water; and soft/sport drink	Telephone interview (CATI) on physical activity included the time spent per day doing organised sport. Proxy interviews for persons under the age of 16.	CATI on reading for pleasure; studying or doing homework; sleeping; and participating in screen-based activities (watching TV, videos or playing video or computer games)	No significant relationship was found between diet quality and maternal employment.	High risk of bias
Koca et al., 2017^(32)^ Turkey	UMIC	Cross sectional study	6–18 years (n = 7116) Random sampling.	Multiple linear regression analysis	Self-reported. Working mother 39.5%	Self-reported semi-quantitative FFQ	Self-reported out-of-school physical activity (activity by the child either alone, in sports clubs, or with family or friends)	–	Children of working mothers are more active.	Low risk of bias
Ben-Shalom, 2010^(33)^ USA	HIC	Cross sectional study	0–18 years +	Becker's model of household production (Becker 1965)	Full-time employment ≥ 35 h worked per week	Parents interview on the HEI and important nutrients	The number of days per week the child gets rapid exercise.	Parents interview on Tv watching hours/week	In married couple family’s food-intake quality decrease with maternal employment, but this association is weaker for single mother families (first study) and children are more likely to get rapid exercise when their mothers work more hours per week (second study).	High risk of bias
Gwozdz et al., 2013^(34)^ Belgium, Cyprus, Estonia, Germany, Hungary, Italy, Spain and Sweden	Both HIC & UMIC (Estonia)	Cross sectional study	5–9 years (n = 7000) Data collected from 390 kindergartens and school.	Multiple regression and quantile regression.	Self-reported employment. Full-time employment ≥ 35 h worked per week: part time employment < 35 h per week and not in paid employment.	Self-administered Youth Healthy Eating Index	Uniaxial accelerometry (Non structured activities)		Maternal employment had negative association with children’s calorie intake and positive to physical activity	Low risk of bias
Datar et al (2014)^(35)^ USA	HIC	Longitudinal study	5th & 8th grade student (n = 20,020)	OLS and IV estimate.	Self-reported hours of work per week.	Interview with food consumption questionnaire	Self-reported regular and vigorous exercise (min 20 min/day/week)	Television viewing	Maternal employment was positively associated with consumption of soda, fast food, physical activity and sedentary behaviour and negatively related to fruits, vegetables and milk consumption.	Low risk of bias
Parker, 2007^(36)^ USA	HIC	Cross sectional study	5–18 years (N = 260)	Cronbach’s Alpha, Pearson correlations and one-way ANOVA.	Self-reported employment status as full time, part time, and not employed	–	Daily step count using pedometer	–	Association of maternal employment and children’s physical activity was not statistically significant.	High risk of bias
Raheeq and Arshad (2020)^(37)^ Pakistan	LMIC	Cross sectional study	5–10 years (n = 250)	Tabular analysis	Self-reported as working and stay at home mothers	–	–	TV watching	Children of working mothers follow the rules about the time duration of watching television more compared to the children of home-maker mothers.	High risk of bias
Ferrari et al. (2016)^(38)^ Brazil	UMIC	Cross sectional study	10 years (n = 328)	Multilevel linear regression model	Self-reported employment as none, less than part time, part time, or full time	FFQ	MVPA using an Actigraph GT3X + accelerometer	TV, video game, and computer time	Children MVPA was negatively associated with maternal employment (mothers who worked part time or less had less MVPA than children whose mothers worked full time).	High risk of bias
Pearson et al. (2009)^(39)^ Australia	HIC	Longitudinal	12–15 years (n = 1884)	Logistic regression and multinominal logistic regression model	Maternal employment = full-time, part-time or not in paid employment	Online survey by FFQ	–	–	Girls of part time employed mothers or not in paid employment had an inverse association with their snack and fast-food consumption.	Low risk of bias
Wijtzes et al. (2014)^(40)^ Netherlands	HIC	Cross sectional study	6 years (n = 4726)	multiple logistic regression	Employment = no paid job, paid job, part-time [< 36 h /week], paid job full time [>_36 hours/week]		Parent-reported children’s sports participation (yes, no) and outdoor play.		Children’s sports participation was negatively associated with maternal unemployment.	Low risk of bias
Lopoo (2007)^(41)^ USA	HIC	Longitudinal	15 years (n = ) the 1996 Survey of Income and Program Participation	Logit and fixed-effects logit models	Mother worked ≤ 30 h and mother worked>30 h.	–	Self-reported after school activities (sports, lesson)	–	A positive relationship between maternal work hours and sports participation.	Low risk of bias
Richards and Duckett (1994)^(42)^ USA	HIC	Longitudinal study	10–13 years (n = 295)	Analysis of variance	Not employed, employed part time (1–34 h/week, employed full time (35 or more hours/week).		Self-reported sports and chores.	Self-reported classwork, homework, TV, socializing, general leisure (e.g., games, reading, extracurricular activities).	Full-time maternal employment was associated with more time doing homework with mothers, less time playing sports, more time watching TV and less time in general leisure and less time in general leisure, while part-time employment was associated with more time doing sports with parents.	High risk of bias

OLS: Ordinary Least Square; FE: Fixed Effect; IV: Instrumental Variable; RE: Random Effect; 2SLS: Two Stage Least Square.

### 2.6 Risk of bias

Risk of bias of the included studies was assessed using a modified version of Cochrane Collaboration tool adopted for observational studies following Higgins et al. (2011), and the JBI Critical Appraisal Checklist was used for the qualitative study. The adapted tool of Cochrane Collaboration has been used in prior studies (Poitras et al., 2016, Prince et al., 2017, Castro et al., 2018). The tool for observational studies focused on six potential sources of bias: selection bias (sampling method), performance bias (measurement of maternal employment), detection bias (measurement of DP, PA, SB), attrition bias (completeness of outcome data), selective reporting bias (selective outcome reporting), and other bias (control for confounding). Each type of bias was marked as “high”, “low”, or “unclear” according to pre-specified criteria. The comprehensive explanation of these criteria is provided in the supplementary document. One reviewer [SA] assessed the risk of bias score while the other reviewers verified these by assessing randomly selected 2 studies each and discussed any conflicting results (initially 83% consistency was attained between reviewers). Further disagreements were resolved through team discussion. The overall risk of bias score was determined by summing the total number of criteria marked as ‘low risk of bias’, ‘high risk of bias’ and ‘unclear risk of bias’ according to the pre-established criteria. The JBI Critical Appraisal Checklist was used to assess the quality of the qualitative study based on study methodology. The corresponding score (out of 10 with 10 the highest) and the JBI Level of Evidence of Meaning (range from 1 to 5 with 5 the lowest) was applied. (Detail documents are available in supplementary files).

## 3 Results

**Fig. 1:**
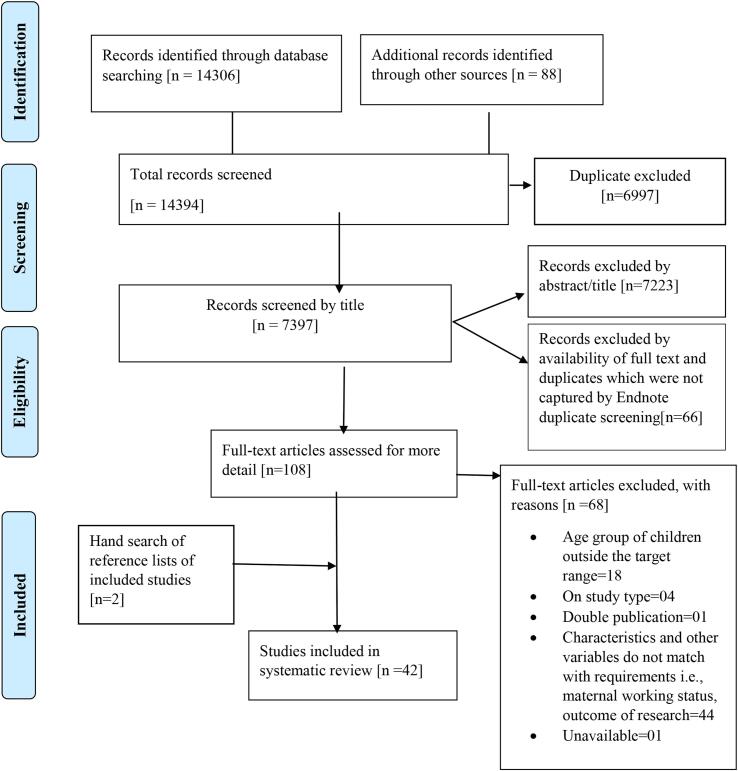
PRISMA flow diagram for selection of studies.

**Fig. 2:**
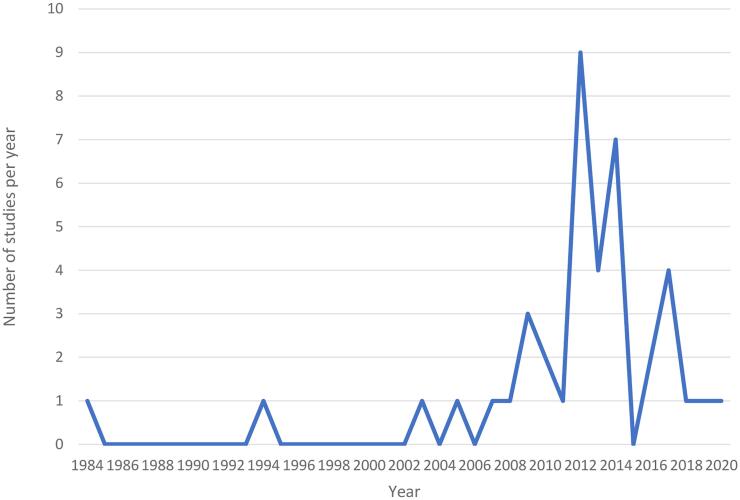
The number of studies on the association of maternal employment to DP, PA and SB published per year.

The search of bibliographic databases yielded 14,306 potentially relevant citations, with a further 88 identified through the secondary backward reference searching. Full text papers were reviewed for 108 studies, of which 68 were excluded. A further two papers were identified from reviewing reference lists of included papers, providing a total of 42 papers for the review, as shown in Fig. 1. The papers were published between 1984 and 2020, with 95.2% published in 2000s (see Fig. 2). Most studies were peer-reviewed journal articles (85.7%), with others being theses (9.5%), conference papers (2.3%), and working papers (2.3%). Results showed an association of maternal employment with all three variables of dietary patterns, physical activity and sedentary behaviour in 9.5% papers, while 59.5% of studies focused on any one of the three variables. The remaining studies (30.9%) included a combination of two variables (dietary pattern-physical activity; dietary pattern-sedentary behaviour or physical activity- sedentary behaviour). This review identified 10 domains for dietary patterns, two for physical activity and four for sedentary behaviour.

### 3.1 Maternal employment and dietary patterns

Among the 42 studies included in the review 26 assessed dietary patterns, with 11 assessing dietary patterns using standard dietary pattern questionnaires, three with 24 h dietary recall, two studies used 3-day food diaries, four used food frequency questionnaires (FFQ), two used a healthy eating index (HEI), two used a youth and adolescent food frequency questionnaire (YAQ) and Global school based student health survey (GSHS) questionnaire, and 2-day food diary each were used by one study. Ten studies used self-reporting or parent reporting measures to assess dietary patterns. The review identified ten domains of dietary patterns, and eight were studied three or more times. Snack’s food including fast food & junk food was the most assessed domain of dietary pattern used in 9 studies followed by family meals assessed in 8 studies. Four studies (Bauer et al., 2012, Datar et al., 2014, Gaina et al., 2009, Meyer, 2016) among nine reported an increase in snack food consumption when the mother was in full-time employment, three studies (Brown et al., 2010, Sweeting and West, 2005, Taylor et al., 2012) reported no association, and two studies (Adbi et al., 2017, Pearson et al., 2009) reported a negative association with employment status of the mother. Family meal (eating together and meals with family members) had a negative relationship with maternal employment in seven (Anderson, 2012, Bauer et al., 2012, Chang and Lee, 2012, Chang, 2012, Gwozdz et al., 2013, Nadia, 2012, Neumark-Sztainer et al., 2003) out of eight papers, with one (Nie and Sousa-Poza, 2014) showing no association. Maternal employment was negatively associated with children’s fruit and vegetables consumption in five studies (Bauer et al., 2012, Datar et al., 2014, Meyer, 2016, Nadia, 2012, Sethi et al., 2014) and one study (Taylor et al., 2012) reported no association. Maternal employment was negatively associated with children’s juice, soda, and soft/sports drinks consumption in one sample (Nadia, 2012), positively in one sample (Datar et al., 2014) and no association in another study (Taylor et al., 2012). Healthy eating habits was negatively related to maternal employment in three studies (Bauer et al., 2012, Nadia, 2012, Sweeting and West, 2005) and positively related in one study (Honajee et al., 2012). Five studies (Chowhan and Stewart, 2014, Fitzsimons and Pongiglione, 2019, Gaina et al., 2009, Nadia, 2012, Shuhaimi and Muniandy, 2012) reported a negative relationship with maternal employment for eating meals regularly.

Dietary quality showed a positive relationship with maternal employment in one study (Touliatos et al., 1984), a negative relationship in two studies (Ben-Shalom, 2010, Li et al., 2012) and was unrelated in another study (Taylor et al., 2012). Maternal employment was negatively associated with children’s energy/calorie, protein, and fat intake in two samples (Gwozdz et al., 2013, Shuhaimi and Muniandy, 2012), and unrelated in one sample (Nie and Sousa-Poza, 2014). The effect of intensity of maternal employment on children’s DP was reported in 8 studies (Bauer et al., 2012, Datar et al., 2014, Fitzsimons and Pongiglione, 2019, Gaina et al., 2009, Li et al., 2012, Meyer, 2016, Nadia, 2012, Sweeting and West, 2005). Four studies (Bauer et al., 2012, Datar et al., 2014, Gaina et al., 2009, Lopez-Arana et al., 2013) reported a positive association of full-time maternal employment with consumption of soda and fast food whereas another four studies found three domains (eating meals regularly, fruits and vegetables consumption, dietary quality) to be negatively associated with full-time maternal employment (Fitzsimons and Pongiglione, 2019, Bauer et al., 2012, Gaina et al., 2009, Lopez-Arana et al., 2013). Dietary patterns and maternal part-time employment were reported in three studies (Fitzsimons and Pongiglione, 2019, Gaina et al., 2009, Sweeting and West, 2005) -positively associated with two domains (snacking and eating dinner regularly) in one study (Gaina et al., 2009) and negatively in two domains (eating breakfast regularly and healthy eating) in two studies (Fitzsimons and Pongiglione, 2019, Sweeting and West, 2005).

In brief, 28 samples reported negative, 9 samples positive, and 9 samples reported no association between children’s dietary patterns and maternal employment. Overall, the associations between maternal employment and children’s dietary patterns showed more adverse than favourable directions.

### 3.2 Maternal employment and physical activity

Nineteen studies (Anderson, 2012, Aniza and Fairuz, 2009, Ben-Shalom, 2010, Chang and Lee, 2012, Chia, 2008, Cho, 2017, Datar et al., 2014, Ferrari et al.,, 2016, Gwozdz et al., 2013, Koca et al., 2017, Lopoo, 2007, Maher et al., 2017, Meyer, 2016, Morrissey et al., 2011, Nie and Sousa-Poza, 2014, Parker, 2007, Richards and Duckett, 1994, Vazquez-Nava et al., 2013, Wijtzes et al., 2014) reported an association between children’s physical activity and maternal employment. Of these 19 studies, over 50% used self-reported methods, 21% used interview and 26.3% used device-based methods to measure physical activity. Three domains of physical activity, MVPA, sports participation and playing with children, were identified, with first two domains studied three or more times. Maternal employment was positively related to children’s MVPA in ten samples (Anderson, 2012, Aniza and Fairuz, 2009, Ben-Shalom, 2010, Chia, 2008, Cho, 2017, Datar et al., 2014, Gwozdz et al., 2013, Koca et al., 2017, Morrissey et al., 2011, Meyer, 2016), negatively in three samples (Ferrari et al.,, 2016, Maher et al., 2017, Vazquez-Nava et al., 2013) and unrelated in two sample (Nie and Sousa-Poza, 2014, Parker, 2007). Maternal employment was positively related to children’s sports participation in one sample (Lopoo, 2007) and negatively in two samples (Richards and Duckett, 1994, Wijtzes et al., 2014). The effect of intensity of maternal employment on children’s physical activity was reported in 3 studies (Ben-Shalom, 2010, Ferrari et al.,, 2016, Richards and Duckett, 1994). One study (Ben-Shalom, 2010) reported a positive association of MVPA with full-time maternal employment whereas part-time maternal employment showed negative association with MVPA in one study (Ferrari et al., 2016). Children’s sports participation was reported negative association in one study (Richards and Duckett, 1994) when mother works full-time. Same study (Richards and Duckett, 1994) reported positive association between maternal part-time employment and playing with children.

Briefly, 11 samples reported positive association whereas 5 samples observed negative association with maternal employment. Overall, working mothers were more likely to have active children.

### 3.3 Maternal employment and sedentary behaviour

Twenty studies (Anderson, 2012, Brown et al., 2010, Chang, 2012, Chia, 2008, Cho, 2017, Richards and Duckett, 1994, Datar et al., 2014, Fitzsimons and Pongiglione, 2019, Ham et al., 2013, Hsin and Felfe, 2014, Maher et al., 2017, Martin et al., 2018, Meyer, 2016, Morrissey et al., 2011, Nadia, 2012, Nie and Sousa-Poza, 2014, Raheeq and Arshad, 2020, Richards and Duckett, 1994, Vazquez-Nava et al., 2013, Ziol-Guest et al., 2013) reported an association between sedentary behaviour and maternal employment. Four domains of sedentary behaviour were identified, with three domains studied three or more times. Watching Television (TV) was the most assessed sedentary behaviour (n = 13). Among the studies assessed TV watching, eight reported a positive association (Datar et al., 2014, Chia, 2008, Chowhan and Stewart, 2014, Fitzsimons and Pongiglione, 2019, Morrissey et al., 2011, Nadia, 2012, Richards and Duckett, 1994, Ziol-Guest et al., 2013), three reported a negative association (Anderson, 2012, Chowhan and Stewart, 2014, Parker, 2007) and two study reported no association (Chang, 2012, Nie and Sousa-Poza, 2014) with maternal employment. Screen time (TV, DVD, video, movie, playing video or computer games) was the second most assessed sedentary behaviour (n = 6). In these studies, five (Cho, 2017, Ham et al., 2013, Martin et al., 2018, Meyer, 2016, Vazquez-Nava et al., 2013) reported a positive association with screen time and maternal employment and one paper (Brown et al., 2010) reported a negative association. Average minutes of sedentary behaviour was assessed in three studies, with two (Maher et al., 2017, Nie and Sousa-Poza, 2014) reporting no association and one (Hsin and Felfe, 2014) a negative association with maternal employment. The effect of intensity of maternal employment on children’s sedentary behaviour was reported in 5 studies (Brown et al., 2010, Fitzsimons and Pongiglione, 2019, Hsin and Felfe, 2014, Meyer, 2016, Nadia, 2012). Three studies (Fitzsimons and Pongiglione, 2019, Meyer, 2016, Nadia, 2012) reported a positive association of full-time maternal employment with two domains (watching TV and screen time) of sedentary behaviour. TV watching was reported both positive (Fitzsimons and Pongiglione, 2019) and negative (Brown et al., 2010) association with part time maternal employment. Average minutes of sedentary behaviour was negatively related with full-time maternal employment in one study (Hsin and Felfe; 2014).

In sum, 13 samples reported positive, 5 samples negative, and 4 samples reported no association between children sedentary behaviour and maternal employment.

### 3.4 Differences in outcome between countries differing in income status

The World Bank’s income classification of countries was used in this review. Based on this classification, 32 of 42 reviewed studies were from HIC, 7 from UMIC, and 3 from LMIC. Table 3 summarizes the associations between maternal employment and dietary patterns, physical activity and sedentary behaviour in high-, upper-middle and lower-middle income countries. The association of dietary patterns, physical activity and sedentary behaviour showed an indeterminate relationship with maternal employment in LMIC and UMIC. Twenty-six studies examined dietary patterns in HICs with the majority from the USA (n = 16). The association between “healthy dietary patterns of children in HIC” were indeterminately related to maternal employment. Physical activity and sedentary behaviour were found to have positive associations with maternal employment in HIC. Eight out of thirteen studies reported positive associations between children’s physical activity and maternal employment. Twenty-one studies reported sedentary behaviour, with 14 positively related to maternal employment.

**Table 2:** Summary of samples showing the associations between maternal employment and different domains of dietary patterns, physical activity, and sedentary behaviour for children (n = 83).

**Domains of Dietary patterns**	**Related to Maternal employment Study no**	**Association (+/−)**	**Unrelated to Maternal employment**	**Number of samples analysed (n)**	**Association**	**Summary code (% of study)**
Snacks food including fast food & junk food	15, 17, 28, **35**, 3 39	+ −	1, 19, 31	n = 9	?	5/9 55.6%
Family meals	6, **17, 22**, 23, 24 30, 34	–	29	n = 8	–	7/8 88%
Fruits & vegetables	**4, 17**, 24, 28, **35**	–	31	n = 6	–	5/6 83.33%
Juice, water, soda, soft/sports drinks	24 **35**	− +	31	n = 3	0	1/3 33.3%
Milk and milk products	**4** **35**	+ −				
Healthy eating habits	7 **17,** 19, 34	+ −		n = 4	–	3/4 75%
Eating meals (breakfast/dinner) regularly	8, **11,** 15, 24, **25**	–	15*	n = 6	–	5/6 62.5%
Dietary quality	14, 18 33	+ −	31	n = 4	?	2/4 50%
Energy/ calorie, protein & fat intake	**25, 34**	–	29	n = 3	–	2/3 66.6%
Eating out at restaurants	**5**	+				
**Domains of Physical activity**						
MVPA	2, 10, 38 9, 20, 21, **26**, 28 30, 32, 33, **34, 35**	− +	29, 36	n = 15	+	10/15 66.6%
Sports participation	40, 42 41	− +		n = 3	–	2/3 66.6%
Playing with their children	**22** 42	− +				
**Domains of Sedentary behaviour**						
Watching TV	8, **11^ϯ^**, 13, 20, 21 24, **35**, 42 **11**, 30, 39	+ −	25, 31	n = 13	+	8/13 61.5%
Screen time (combining TV, DVD, video or movie, playing video/computer games)	1 9, 12, 27, 28	− +	2	n = 6	+	4/6 66.7%
Average minutes of sedentary behaviour (i.e., < 100 counts per minute)	**16**	–	10, 29	n = 3	0	1/3 33.3%
Other sitting activities (reading for pleasure, writing, playing musical instrument)	**42**	+	29			

*Eating breakfast shows ^0^ & dinner shows ^–^ association.

Ϯ Maternal hours worked, and weeks worked had different outcome.

**Table 3:** Summary of studies showing the association between maternal employment and dietary patterns, physical activity and sedentary behaviour based on Lower-middle income, Upper-middle income and High-income countries.

**Type of country (number of studies from each country)**		**Study no**	Association	**Summary code** (% of study)
LMIC (7.1%) (India = 2, Pakistan = 1)	DP	11, 12^−^		
	PA	–		
	SB	37^−^		
UMIC (16.7%) (China = 1, Mexico = 1, Malaysia = 2, Mauritius = 1 Turkey = 1, Brazil = 1)	DP	7^+^ 25^−^ 29^0^	?	1/3 33.3%
	PA	26^+^, 32^+^ 29^0^, 38^−^	?	2/4 50%
	SB	2^+^ 29^0^	?	
HIC (76.2%) (USA = 16, UK = 1, Canada = 2, Australia = 4, Japan = 1, South Korea = 4, Scotland = 1, Germany = 1, Netherlands = 1 6 European countries (Belgium, Cyprus, Estonia, Germany, Hungary, Italy, Spain, and Sweden) = 1	DP	5^+^, 14^+^, 15^+^, 18^+^, 28^+^, 35^+^ 6^−^, 8^−^, 11^−^, 15^−^, 17^−^ 19^−^, 22^–^, 23^–^, 24^−^, 28^−^, 30^−^, 33^–^, 34^−^, 35^−^, 39^−^, 1^0^, 15^0^, 19^0^, 29^0^, 31^0^.	?	15/26 57.7%
	PA	9^+^, 20^+^, 21^+^, 28^+^, 30^+^, 34^+^, 35^+^, 41^+^ 10^–^, 22^–^, 40^−^, 42^−^ 36^0^	+	8/13 61.5%
	SB	2^+^, 8^+^, 9^+^, 11^+^, 12^+^, 13^+^, 16^+^, 20^+^, 21^+^, 24^+^, 27^+^, 28^+^, 35^+^, 42^+^ 1^−^, 11^−^, 16^−^, 30^−^ 10^0^, 23^0^, 29^0^	+	14/21 66.6%

### 3.5 Risk of bias assessment

Across all studies, 57.1% (n = 24) had high risk of bias score. The remaining 40.5% studies (n = 17) were classified as low risk of bias score, while 2.4% (n = 1) had unclear risk of bias. Concerning each criterion of risk of bias for observational studies, 56.1% of studies (n = 23) used probability sampling methods and were hence identified as low risk of bias. The majority of the studies (70.8%) had high risk of performance bias as most cases of ‘maternal employment’ were measured using non-validated tools. Similar to performance bias, over half of detection bias (51.2%) had high risk for using non-validated measurements for dietary patterns, physical activity and sedentary behaviour. Over one third of studies (41.5%; n = 17) did not manage < 20% of missing data and were marked as high risk for attrition bias. One third of studies (34.1%; n = 14) did not provide any reasons for missing data and were identified as unclear risk of attrition bias. Reporting bias is low for two-thirds of the studies (70.7%; n = 29) and 17.1% (n = 7) had high risk of reporting bias. Nearly two-thirds of studies (63.1%; n = 26) reported about the statistical methods to control for potential confounding factors, and hence were coded as low risk of bias; and 22% (n = 9) did not provide sufficient information regarding confounding factors. Detailed risk of bias results is available in the supplementary material.

Effect size is measured for individual studies. Effect sizes ranged from −0.08. to 3.8. Majority of studies fail to produce medium to large effect size.

## 4 Discussion

The aim of this systematic review was to determine whether maternal employment was associated with children’s health behaviours, specifically dietary patterns, physical activity, and sedentary behaviour. A wide range of domains for these behaviours among children within HIC and LMIC were identified. These domains were assessed using various tools and the association with maternal employment was varied. The review shows that the number of studies on these lifestyle variables among children related to maternal employment has expanded in the last two decades, with two studies between 1980s and 1990s, and all remaining studies published in the 2000s.

Results showed that maternal employment was inversely associated with children’s family meals (Anderson, 2012, Bauer et al., 2012, Chang and Lee, 2012, Chang, 2012, Gwozdz et al., 2013, Nadia, 2012, Neumark-Sztainer et al., 2003), fruits and vegetables consumption (Bauer et al., 2012, Datar et al., 2014, Meyer, 2016, Nadia, 2012, Sethi et al., 2014), healthy eating habits (Bauer et al., 2012, Gwozdz et al., 2013, Sweeting and West, 2005), eating meals regularly (Chowhan and Stewart, 2014, Fitzsimons and Pongiglione, 2019, Gaina et al., 2009, Nadia, 2012, Shuhaimi and Muniandy, 2012), and energy/calorie, protein, and fat intake (Gwozdz et al., 2013, Shuhaimi and Muniandy, 2012). In dual income families it is expected that maternal employment may allow families to spend more on healthy foods (Lowery et al., 2019), however, employment is likely to create a time constraint for meal related behaviours (Devine et al., 2003, Jabs et al., 2007). Studies show that employed mothers spent significantly less time in meal preparation (Beshara et al., 2010, Cutler et al., 2003) and consume more meals prepared away from home (Kant and Graubard, 2004). Their children are less likely to eat fruits and vegetables (Hawkins et al., 2009) and more likely to skip meals and have unfixed snacking time (Watanabe et al., 2011). In addition to this, employed mothers have less time to supervise their children, which creates opportunities for poorer diets (Datar et al., 2014). Hence, time pressures for employed mothers may have a greater detrimental effect on children’s food intake patterns as reflected in fewer family meals, less consumption of fruits and vegetables, less healthy eating habits, and greater irregularity or ‘skipping’ of meals (breakfast and dinner). Juice, water, soda, soft/sports drinks were found to have no association with maternal employment (Datar et al., 2014, Nadia, 2012, Taylor et al., 2012). In this review an indeterminate association with maternal employment was found in children’s consumption of snack food, including fast food and junk food (Adbi et al., 2017, Brown et al., 2010, Datar et al., 2014, Gaina et al., 2009, Meyer, 2016, Sweeting and West, 2005, Taylor et al., 2012), and dietary quality (Ben-Shalom, 2010, Li et al., 2012, Taylor et al., 2012, Touliatos et al., 1984). Overall, further investigations are needed to determine more definite conclusions for those domains (e.g., milk and milk products, eating out at restaurant) that had indeterminate outcome because of the small number of studies available. MVPA was positively associated with maternal employment (Anderson, 2012, Aniza and Fairuz, 2009, Ben-Shalom, 2010, Chia, 2008, Cho, 2017, Datar et al., 2014, Gwozdz et al., 2013, Koca et al., 2017, Meyer, 2016, Morrissey et al., 2011) whereas sports participation showed a negative association (Lopoo, 2007, Wijtzes et al., 2014) in the current review. Overall, it is indicated that children of working mothers were sufficiently physically active. This finding may seem somewhat contradictory. Working mothers bear a double or triple burden of responsibilities at home and at work (Bond and Sales, 2001), and thus may lack sufficient remaining time and energy to more fully supervise and actively engage with their children (Cawley and Liu, 2012, Fertig et al., 2009). However, monetary support from employed mothers may lead a better quality of life. There is evidence that higher socioeconomic status (SES) of families provides more opportunities for their children to do more activities, some of which are physical activities (Park and Kim, 2008, Stalsberg and Pedersen, 2010), and they could financially support the enrollment of their children into organized physical activity such as active sports clubs (Datar et al., 2014, Kantomaa et al., 2007).

In terms of TV watching in children as a sedentary behaviour, most studies reported positive associations with maternal employment (Chia, 2008, Chowhan and Stewart, 2014, Datar et al., 2014, Fitzsimons and Pongiglione, 2019, Nadia, 2012, Richards and Duckett, 1994, Sweeting and West, 2005, Ziol-Guest et al., 2013). TV viewing has become a preferred leisure time activity of children during unsupervised time (Datar et al., 2014) and the reason may be the unavailability of outdoor facilities or due to safety reasons. Existing literature support positive associations between maternal employment and TV viewing of children (Fertig et al., 2009). Screen time (TV, DVD, video, or movie, playing video/computer games) indicated a positive relationship with maternal employment (Cho, 2017, Ham et al., 2013, Martin et al., 2018, Meyer, 2016). Children and adolescent’s increasing exposure to screen-based activities are evident in other reviews (Thomas et al., 2019). Results show that children of employed mothers were physically active, but at the same time, children spent more time on sedentary pursuits. While this may appear contradictory, it has been shown that physical activity can be independent of how much time children spend in sedentary behaviours over the day (Marshall et al., 2004, Pearson et al., 2014, Sallis et al., 2000). For example, a child can indulge in high levels of MVPA but also in sedentary screen time. Within a 24-h day, time can be displaced to lighter forms of physical activity or sleep.

### 4.1 Strength and limitations of the study

Applying a comprehensive and systematic approach, this review included a detailed summary and critical narrative synthesis of 42 published papers. Additional strengths of the review are the inclusion of all study designs as well as all type of measures of dietary patterns, physical activity and sedentary behaviour. Furthermore, this review included multiple domains of dietary patterns, physical activity and sedentary behaviour.

This systematic review has some limitations. Although we tried to identify as many studies as possible, we may have inadvertently missed some eligible studies due to limited search strategy and beyond published English language studies. Furthermore, a majority of the studies were cross-sectional, thus conclusions regarding causality of association are not possible. Although device-based measures of physical activity and sedentary behaviour are more reliable, only a small number of studies used them. Most studies used self-report or maternal-report data, hence contributing to the possibility of reporting bias. Risk of bias among included studies were relatively high since many studies did not report on the reliability and validity of measures used to assess maternal employment and dietary patterns, physical activity and sedentary behaviour. The diverse nature of measures and outcomes prevented the use of *meta*-analysis. Conceptually similar domains were combined which may also narrow down the depth of analysis and generalizability of these findings. Though, multivariate tests are more accurate, use of univariate test for assessing statistical significance is a limitation of this study. Finally, most of the studies are from HIC, hence findings may not be similar in the context of LMIC.

## 5 Conclusion

To our knowledge, this is the first systematic review that summarises the evidence for links between dietary patterns, physical activity, and sedentary behaviour of children with the employment status of their mothers. Findings suggest that maternal employment was associated with poor dietary patterns but more physical activity and more time on sedentary activities. The latter were particularly for TV viewing and other screen-based activities of children. These findings provide an indication of how maternal employment may increase the risk of childhood obesity. We also identified a lack of validated measurers of dietary patterns and few studies using device-based assessment of physical activity and sedentary behaviour. The findings of this systematic review have important implications in the context of growing participation of women in the labour force. Considering that the employment and economic activity of women will continue to increase in the future, interventions should support employed mothers with an aim to promote healthier children’s dietary patterns and decrease sedentary time. Little can be deduced from the inadequately studied domains (for example-milk and milk products, eating out at restaurant, mothers playing with their children, sitting activities (reading-writing, playing musical instruments)), hence future studies need to focus on these domains of dietary patterns, physical activity and sedentary behaviour. Domains that have inconsistent results also require further testing. In addition to this, future research needs to pay attention to UMIC and LMIC because research related to maternal employment and children’s lifestyle variables are scarce in those countries. Moreover, the use of device-based measures of physical activity and sedentary behaviour are needed in order to produce accurate estimates of total time spent in physical activity and sedentary behaviour.

## 6 Financial support

This research did not receive any financial support from any organization.

## 7 Ethics approval

N/A

## 8 Consent

N/A

## 9 Availability of data and materials

Data and other material will be provided as supplementary document.

## Authors contributions

All authors contributed to the planning and design of the systematic review. The corresponding author performed the literature searches, full text screening, data extraction and analysed, and wrote the first draft of the review. The other authors contributed to the screening of the studies for eligibility, to the risk of bias assessment of studies. All authors reviewed, edited, and approved the final draft. The corresponding author attests that all listed authors meet authorship criteria and that no others meeting the criteria have been omitted.

## Declaration of Competing Interest

The authors declare that they have no known competing financial interests or personal relationships that could have appeared to influence the work reported in this paper.

## References

[b0005] Adbi A., Faizi N., Chatterjee C. (2017). Analysing the lazy mother argument inspired by the maggi controversy: Evidence from junk food intake in India. Econ. Polit. Weekly.

[b0010] Anderson P.M. (2012). Parental employment, family routines and childhood obesity. Econ. Hum. Biol..

[b0015] Anderson P.M., Butcher K.F., Levine P.B. (2003). Maternal employment and overweight children. J. Health Econ..

[b0020] Aniza I., Fairuz M.R. (2009). Factors influencing physical activity level among secondary school adolescents in Petaling District, Selangor. Med. J. Malaysia.

[b0025] Barnett T.A., Kelly A.S., Young D.R., Perry C.K., Pratt C.A., Edwards N.M., Rao G., Vos M.B. (2018). Sedentary Behaviors in Today’s Youth: Approaches to the Prevention and Management of Childhood Obesity: A Scientific Statement from the American Heart Association. Circulation.

[b0030] Baten J., Böhm A. (2010). Children's Height and Parental Unemployment: A Large-Scale Anthropometric Study on Eastern Germany, 1994–2006. German Econ. Rev..

[b0035] Bauer K.W., Hearst M.O., Escoto K., Berge J.M., Neumark-Sztainer D. (2012). Parental employment and work-family stress: associations with family food environments. Soc. Sci. Med..

[b0040] Ben-Shalom, Y., 2010. *Maternal employment, household nutrition and obesity. (Ph.D.), T*he Johns Hopkins University, Ann Arbor.

[b0045] Beshara M., Hutchinson A., Wilson C. (2010). Preparing meals under time stress. The experience of working mothers. Appetite.

[b0050] Bianchi S.M. (2000). Maternal employment and time with children: Dramatic change or surprising continuity?. Demography.

[b0055] Biddle S.J.H., Gorely T., Stensel D.J. (2004). Health-enhancing physical activity and sedentary behaviour in children and adolescents. J. Sports Sci..

[b0060] Bond S., Sales J. (2001). Household work in the UK: an analysis of the British Household Panel Survey 1994. Work Employ Soc..

[b0065] Brown J.E., Broom D.H., Nicholson J.M., Bittman M. (2010). Do working mothers raise couch potato kids? Maternal employment and children's lifestyle behaviours and weight in early childhood. Soc. Sci. Med..

[b0070] Castro O., Bennie J., Vergeer I., Bosselut G., Biddle S.J.H. (2018). Correlates of sedentary behaviour in university students: A systematic review. Prev. Med..

[b0075] Cawley J., Liu F. (2012). Maternal employment and childhood obesity: A search for mechanisms in time use data. Econ. Hum. Biol..

[b0080] Chang Y., Lee S. (2012). Does Maternal Employment Affect Parental Time Allocated to Children's Food Consumption and Physical Activity? Evidence from the Korean Time Use Survey. Int. J. Hum. Ecol..

[b0085] Chang Y.-J. (2012).

[b0090] Chia Y.F. (2008). Maternal labour supply and childhood obesity in Canada: evidence from the NLSCY. Canad. J. Econ./Revue canadienne d'économique.

[b0095] Cho Y. (2017). Maternal work hours and adolescents’ body weight in South Korea. Asian Popul. Stud..

[b0100] Chowhan J., Stewart J.M. (2014). While mothers work do children shirk? Determinants of youth obesity. Appl. Econ. Perspect. Policy.

[b0105] Cutler D.M., Glaeser E.L., Shapiro J.M. (2003). Why have Americans become more obese?. J. Econ. Perspect..

[b0110] Das P., Horton R. (2012). Rethinking our approach to physical activity. Lancet.

[b0115] Datar A., Nicosia N., Shier V. (2014). Maternal work and children's diet, activity, and obesity. Soc. Sci. Med..

[b0120] Devine, C.M., Connors, M.M., Sobal, J.C.A., 2003. Sandwiching it in: spillover of work onto food choices and family roles in low-and moderate-income urban households. Social Sci. Med., 56(3), 617–630. 10.1016/s0277-9536(02)00058-8 12570978

[b0125] Dodzin S., Vamvakidis A. (2004). Trade and industrialization in developing economies. J. Dev. Econ..

[b0130] Duch H., Fisher E.M., Ensari I., Harrington A. (2013). Screen time use in children under 3 years old: a systematic review of correlates. Int. J. Behav. Nutr. Phys. Activity.

[bib416] Ferrari G.L., V., Barreira, T. V., Tudor-Locke, C., Katzmarzyk, P. T., & Fisberg, M. (2016). Correlates of Moderate-to-Vigorous Physical Activity in Brazilian Children. J. Phys. Activity and Health.

[b0135] Fertig A., Glomm G., Tchernis R. (2009). The connection between maternal employment and childhood obesity: Inspecting the mechanisms. Rev. Econ. Household.

[b0140] Fitzsimons E., Pongiglione B. (2019). The impact of maternal employment on children's weight: Evidence from the UK. SSM - Population Health.

[b0145] Gaina A., Sekine M., Chandola T., Marmot M., Kagamimori S. (2009). Mother employment status and nutritional patterns in Japanese junior high schoolchildren. Int. J. Obes..

[b0150] Gwozdz W., Sousa-Poza A., Reisch L.A., Ahrens W., Eiben G., Fernandéz-Alvira J.M., Hadjigeorgiou C., De Henauw S., Kovács E., Lauria F., Veidebaum T., Williams G., Bammann K. (2013). Maternal employment and childhood obesity – A European perspective. J. Health Econ..

[b0155] Ham O.K., Sung K.M., Kim H.K. (2013). Factors Associated with Screen Time Among School-Age Children in Korea. J. School Nurs..

[b0160] Hawkins S.S., Cole T.J., Law C. (2009). Examining the relationship between maternal employment and health behaviours in 5-year-old British children. J. Epidemiol. Community Health.

[b0165] Hawkins S.S., Cole T.J., Law C. (2008). Maternal employment and early childhood overweight: findings from the UK Millennium Cohort Study. Int. J. Obes..

[b0170] Higgins, J. P. T., Altman, D. G., Gøtzsche, P. C., Jüni, P., Moher, D., Oxman, A. D., . . . Sterne, J. A. C. (2011). The Cochrane Collaboration’s tool for assessing risk of bias in randomised trials. Bmj, 343, https://doi.org/10.1136/bmj.d5928. 10.1136/bmj.d5928 PMC3196245 22008217

[b0175] Honajee K., Mahomoodally F.M., Subratty A.H., Ramasawmy D. (2012). Is parenting style and sociodemographic status of parents related to children's healthy eating activity in a multicultural society like mauritius?. Asian J. Clin. Nutr..

[b0180] Hoyos Cillero I., Jago R. (2010). Systematic review of correlates of screen-viewing among young children. Prev. Med..

[b0185] Hsin A., Felfe C. (2014). When Does Time Matter? Maternal Employment, Children’s Time With Parents, and Child Development. Demography.

[b0190] Jabs J., Devine C.M., Bisogni C.A., Farrell T.J., Jastran M., Wethington E. (2007). Trying to find the quickest way: employed mothers’ constructions of time for food. J. Nutr. Educ. Behav..

[bib417] Kant A., Graubard B. (2004). Eating out in America, 1987–2000: trends and nutritional correlates. Prev. Med..

[b0200] Kantomaa M.T., Tammelin T.H., Näyhä S., Taanila A.M. (2007). Adolescents' physical activity in relation to family income and parents' education. Prev. Med..

[b0205] Kelder S.H., Perry C.L., Klepp K.I., Lytle L.L. (1994). Longitudinal tracking of adolescent smoking, physical activity, and food choice behaviors. Am. J. Public Health.

[b0210] Koca T., Akcam M., Serdaroglu F., Dereci S. (2017). Breakfast habits, dairy product consumption, physical activity, and their associations with body mass index in children aged 6–18. Eur. J. Pediatr..

[b0215] Lee I.M., Shiroma E.J., Lobelo F., Puska P., Blair S.N., Katzmarzyk P.T. (2012). Effect of physical inactivity on major non-communicable diseases worldwide: an analysis of burden of disease and life expectancy. Lancet.

[b0220] Li J., O'Sullivan T., Johnson S., Stanley F., Oddy W. (2012). Maternal work hours in early to middle childhood link to later adolescent diet quality. Public Health Nutr..

[b0225] Lopez-Arana S., Avendano M., van Lenthe F., Burdorf A. (2013). Trends in overweight among women differ by occupational class: results from 33 low- and middle-income countries in the period 1992–2009. Int. J. Obes..

[b0230] Lowery, C., Oddo, V., Hurley, K., Jones-Smith, J., Gittelsohn, J., Ponce, S. D., & Black, M. (2019). Maternal Employment and Children's Dietary Diversity Scores in Southwestern Guatemala (P10-039-19). Curr. Dev. Nutr., 3(Supplement_1), nzz034. P010-039-019.

[bib418] Lopoo L.M. (2007). While the cat’s away, do the mice play? Maternal employment and the after-school activities of adolescents. Soc. Sci. Quart..

[b0235] Maher J.P., Ra C., O'Connor S.G., Belcher B.R., Leventhal A., Margolin G., Dunton G.F. (2017). Associations between Maternal Mental Health and Well-being and Physical Activity and Sedentary Behavior in Children. J. Dev. Behav. Pediatr..

[b0240] Marshall S.J., Biddle S.J.H., Gorely T., Cameron N., Murdey I. (2004). Relationships between media use, body fatness and physical activity in children and youth: a meta-analysis. Int. J. Obes..

[b0245] Martin M.A., Lippert A.M., Chandler K.D., Lemmon M. (2018). Does mothers’ employment affect adolescents’ weight and activity levels? Improving our empirical estimates. SSM - Population Health.

[b0250] Mech P., Hooley M., Skouteris H., Williams J. (2016). Parent-related mechanisms underlying the social gradient of childhood overweight and obesity: a systematic review. Child Care Health Dev..

[b0255] Meyer S.C. (2016). Maternal employment and childhood overweight in Germany. Econ. Hum. Biol..

[b0260] Moher D., Liberati A., Tetzlaff J., Altman D.G. (2009). Preferred reporting items for systematic reviews and meta-analyses: the PRISMA statement. PLoS Med..

[b0265] Morrissey T.W., Dunifon R.E., Kalil A. (2011). Maternal Employment, Work Schedules, and Children’s Body Mass Index. Child Dev..

[b0270] Mu M., Xu L.-F., Hu D., Wu J., Bai M.-J. (2017). Dietary Patterns and Overweight/Obesity: A Review Article. Iran. J. Public Health.

[b0275] Nadia, Y., 2012. Maternal Employment and Direct Causes of Childhood Obesity. (Master of Applied Economics), The University of Minnesota.

[b0280] Neumark-Sztainer D., Hannan P.J., Story M., Croll J., Perry C. (2003). Family meal patterns: Associations with sociodemographic characteristics and improved dietary intake among adolescents. J. Am. Diet. Assoc..

[b0285] Nie P., Sousa-Poza A. (2014). Maternal employment and childhood obesity in China: evidence from the China Health and Nutrition Survey. Appl. Econ..

[b0290] Park Hyoungsook, Kim Namhee (2008). Predicting factors of physical activity in adolescents: A systematic review. Asian Nurs. Res. (Korean Soc. Nurs. Sci.).

[b0295] Park S., Kang J.H., Lawrence R., Gittelsohn J. (2014). Environmental Influences on Youth Eating Habits: Insights From Parents and Teachers In South Korea. Ecol. Food Nutr..

[b0300] Parker, M.S., 2007. The Relationship Between Maternal Employment and Children's Physical Activity. (MS Theses and Dissertations. 1097), Brigham Young University.

[b0305] Pearson N., MacFarlane A., Crawford D., Biddle S. (2009). Family circumstance and adolescent dietary behaviours. Appetite.

[b0310] Pearson N., Braithwaite R.E., Biddle S.J.H., Sluijs E.M.F., Atkin A.J. (2014). Associations between sedentary behaviour and physical activity in children and adolescents: a meta-analysis. Obes. Rev..

[b0315] Poitras Veronica Joan, Gray Casey Ellen, Borghese Michael M., Carson Valerie, Chaput Jean-Philippe, Janssen Ian, Katzmarzyk Peter T., Pate Russell R., Connor Gorber Sarah, Kho Michelle E., Sampson Margaret, Tremblay Mark S. (2016). Systematic review of the relationships between objectively measured physical activity and health indicators in school-aged children and youth. Appl. Physiol. Nutr. Metab..

[b0320] Prince S.A., Reed J.L., McFetridge C., Tremblay M.S., Reid R.D. (2017). Correlates of sedentary behaviour in adults: a systematic review. Obes. Rev..

[bib420] Raheeq W., Arshad M. (2020). Media exposure among the children of working and non-working mothers in Pakistani urban society. Pakistan J. Appl. Soc. Sci..

[b0325] Raynor Hollie A., Bond Dale S., Freedson Patty S., Sisson Susan B. (2012). Sedentary Behaviors, Weight, and Health and Disease Risks. J. Obes..

[b0330] Rennie K.L., Johnson L., Jebb S.A. (2005). Behavioural determinants of obesity. Best Pract. Res. Clin. Endocrinol. Metab..

[bib419] Richards H.M., Duckett E. (1994). The Relationship of Maternal Employment to Early Adolescent Daily Experience with and Without Parents. Child Develop..

[b0335] Sallis J.F., Prochaska J.J., Taylor W.C. (2000). A review of correlates of physical activity of children and adolescents. Med. Sci. Sports Exerc..

[b0340] Sethi D., Sehgal S., Mehta D. (2014). Maternal employment and children's nutrition-a study from Haryana. Ann. Agri-Bio Res..

[b0345] Savage J.S., Fisher J.O., Birch L.L. (2007). Parental Influence on Eating Behavior: Conception to Adolescence. J. Law Med. Ethics.

[b0350] Shrewsbury V., Wardle J. (2008). Socioeconomic status and adiposity in childhood: a systematic review of cross-sectional studies 1990–2005. Obesity (Silver Spring).

[b0355] Shuhaimi Farhanah, Muniandy Naleena Devi (2012). The association of maternal employment status on nutritional status among children in selected kindergartens in Selangor, Malaysia. Asian J. Clin. Nutr..

[b0360] Stalsberg R., Pedersen A.V. (2010). Effects of socioeconomic status on the physical activity in adolescents: a systematic review of the evidence. Scand. J. Med. Sci. Sports.

[b0365] Sweeting H., West P. (2005). Dietary habits and children's family lives. J. Hum. Nutr. Diet..

[b0370] Tammelin, R., Yang, X., Leskinen, E., Kankaanpaa, A., Hirvensalo, M., Tammelin, T., Raitakari, O., 2014. Tracking of physical activity from early childhood through youth into adulthood. Med. Sci. Sports Exerc. 46(5), 955–962. 10.1249/MSS.0000000000000181 24121247

[b0375] Taylor, A.W., Winefield, H., Kettler, L., Roberts, R., Gill, T.K., 2012. A population study of 5 to15 year olds: Full time maternal employment not associated with high BMI. The importance of screen-based activity, reading for pleasure and sleep duration in children's BMI. Maternal Child Health J. 16(3), 587–599. https://doi.org/:10.1007/s10995-011-0792-y. 10.1007/s10995-011-0792-y PMC3304066 21505779

[b0380] Thomas G., Bennie J.A., De Cocker K., Castro O., Biddle S.J. (2019). A descriptive epidemiology of screen-based devices by children and adolescents: a scoping review of 130 surveillance studies since 2000. Child Indic. Res..

[b0385] Touliatos J., Lindholm B.W., Wenberg M.F., Ryan M. (1984). Family and child correlates of nutrition knowledge and dietary quality in 10–13 year olds. J. Sch. Health.

[b0390] Tremblay Mark S., Aubert Salomé, Barnes Joel D., Saunders Travis J., Carson Valerie, Latimer-Cheung Amy E., Chastin Sebastien F.M., Altenburg Teatske M., Chinapaw Mai J.M. (2017). Sedentary Behavior Research Network (SBRN) – Terminology Consensus Project process and outcome. Int. J. Behav. Nutr. Phys. Activity.

[b0395] Vazquez-Nava F., Trevino-Garcia-Manzo N., Vazquez-Rodriguez C.F., Vazquez-Rodriguez E.M. (2013). Association between family structure, maternal education level, and maternal employment with sedentary lifestyle in primary school-age children. J. Pediatr. (Rio J).

[b0400] Waddell G., Burton A. (2006).

[b0405] Watanabe E., Lee J.S., Kawakubo K. (2011). Associations of maternal employment and three-generation families with pre-school children's overweight and obesity in Japan. Int. J. Obes..

[bib421] Wijtzes A.I., Jansen W., Bouthoorn, S. H., Pot, N., Hofman, A., Jaddoe, V. W., & Raat, H. (2014). Social inequalities in young children’s sports participation and outdoor play. Int. J. Behav. Nutr. Phys. Activity.

[b0410] Wolin K.Y., Carson K., Colditz G.A. (2010). Obesity and cancer. Oncologist.

[b0415] Ziol-Guest Kathleen M., Dunifon Rachel E., Kalil Ariel (2013). Parental employment and children's body weight: Mothers, others, and mechanisms. Soc. Sci. Med..

[bib422] (2020). http://www.who.int/dietphysicalactivity/childhood/en/.

[bib423] (2020). https://data.worldbank.org/indicator/SL.TLF.CACT.FE.ZS.

