# Supplementary material for: Dietary habits, physical activity, and sedentary behaviour of children of employed mothers: A systematic review
Source: Prev Med Rep. 2021 Oct 22;24:101607. doi: 10.1016/j.pmedr.2021.101607 (PMC8683879; doi:10.1016/j.pmedr.2021.101607)
Supplement: Supplementary data 2 [file mmc2.docx]

**Title: Dietary habits, physical activity, and sedentary behaviour of children of employed mothers: a systematic review**

**Authors**:

**Risk of bias summary across selected studies**

A composite risk of bias score for each study was then calculated by summing the total number of criteria:

Low risk of bias: When three or more of the six risk of bias criteria were met

High risk of bias: When less than three of the six risk of bias criteria were met

Unclear risk of bias: When four or more criteria presented an unclear risk of bias due to incomplete reporting.

| **Study** | **Selection bias** | **Performance bias** | **Detection bias** | **Attrition bias** | **Selective reporting bias** | **Other bias: Confounding** | **Risk of bias score** |
| --- | --- | --- | --- | --- | --- | --- | --- |
| Brown, et al. (2010) | **Low risk of bias** | **High risk of bias** | **High risk of bias** | **Low risk of bias** | **Low risk of bias** | **Low risk of bias** | **Low risk of bias** |
| Vazquez-Nava et al.(2013) | **Low risk of bias** | **High risk of bias** | **High risk of bias** | **Unclear risk of bias** | **High risk of bias** | **Unclear risk of bias** | **High risk of bias** |
| Adbi, A et al.(2017) | **Unclear risk of bias** | **Unclear risk of bias** | **Low risk of bias** | **Unclear risk of bias** | **Low risk of bias** | **Low risk of bias** | **Low risk of bias** |
| Sethi, D. et al. (2014). | **Unclear risk of bias** | **High risk of bias** | **High risk of bias** | **Unclear risk of bias** | **Low risk of bias** | **High risk of bias** | **High risk of bias** |
| Park, S., et al. (2014) |  |  |  |  |  |  | **Low risk of bias** |
| Neumark-Sztainer, D., et al. (2003) | **Unclear risk of bias** | **High risk of bias** | **Low risk of bias** | **High risk of bias** | **High risk of bias** | **Low risk of bias** | **High risk of bias** |
| Honajee, K. et al. (2012) | **Unclear risk of bias** | **Unclear risk of bias** | **Low risk of bias** | **Unclear risk of bias** | **High risk of bias** | **Unclear risk of bias** | **Unclear risk of bias** |
| Fitzsimons, E. et al. (2019) | **Low risk of bias** | **High risk of bias** | **Unclear risk of bias** | **High risk of bias** | **Low risk of bias** | **Low risk of bias** | **High risk of bias** |
| Cho, Y. (2017) | **Low risk of bias** | **Low risk of bias** | **High risk of bias** | **Low risk of bias** | **Low risk of bias** | **High risk of bias** | **Low risk of bias** |
| Maher, J. P., et al. (2017) | **Unclear risk of bias** | **Unclear risk of bias** | **Low risk of bias** | **High risk of bias** | **Low risk of bias** | **Unclear risk of bias** | **High risk of bias** |
| Chowhan, J., et al. (2014). | **Unclear risk of bias** | **High risk of bias** | **Low risk of bias** | **Low risk of bias** | **Low risk of bias** | **Low risk of bias** | **Low risk of bias** |
| Ham, O. K., et al (2013). | **High risk of bias** | **Unclear risk of bias** | **Low risk of bias** | **Unclear risk of bias** | **High risk of bias** | **Unclear risk of bias** | **High risk of bias** |
| Ziol-Guest et al., (2013). | **Low risk of bias** | **Low risk of bias** | **Low risk of bias** | **Low risk of bias** | **Unclear risk of bias** | **Low risk of bias** | **Low risk of bias** |
| Touliatos, J., et al. (1984) | **Unclear risk of bias** | **High risk of bias** | **Low risk of bias** | **Unclear risk of bias** | **Low risk of bias** | **High risk of bias** | **High risk of bias** |
| Gaina, A., et al. (2009). | **Unclear risk of bias** | **High risk of bias** | **High risk of bias** | **High risk of bias** | **High risk of bias** | **Low risk of bias** | **High risk of bias** |
| Hsin, A., & Felfe, C. (2014). | **Unclear risk of bias** | **High risk of bias** | **Low risk of bias** | **Low risk of bias** | **Unclear risk of bias** | **Low risk of bias** | **Low risk of bias** |
| Bauer, K. W., et al. D. (2012). | **Unclear risk of bias** | **Low risk of bias** | **Low risk of bias** | **High risk of bias** | **Unclear risk of bias** | **Low risk of bias** | **Low risk of bias** |
| Li, J., et al. (2012). | **Low risk of bias** | **High risk of bias** | **High risk of bias** | **High risk of bias** | **Low risk of bias** | **Low risk of bias** | **High risk of bias** |
| Sweeting, H., & West, P. (2005). | **Unclear risk of bias** | **Unclear risk of bias** | **High risk of bias** | **High risk of bias** | **Low risk of bias** | **Low risk of bias** | **High risk of bias** |
| Morrissey, T. W., et al. (2011) | **Low risk of bias** | **High risk of bias** | **Unclear risk of bias** | **High risk of bias** | **Low risk of bias** | **Low risk of bias** | **High risk of bias** |
| Chia, Y. F. (2008) | **High risk of bias** | **High risk of bias** | **Low risk of bias** | **Unclear risk of bias** | **Low risk of bias** | **Low risk of bias** | **High risk of bias** |
| Chang, Y., & Lee, S. (2012). | **Low risk of bias** | **Unclear risk of bias** | **High risk of bias** | **Unclear risk of bias** | **Low risk of bias** | **Low risk of bias** | **Low risk of bias** |
| Chang, Y.-J. (2012). | **Low risk of bias** | **High risk of bias** | **High risk of bias** | **Unclear risk of bias** | **Low risk of bias** | **Low risk of bias** | **High risk of bias** |
| Nadia, Y. (2012) | **Unclear risk of bias** | **High risk of bias** | **High risk of bias** | **Unclear risk of bias** | **Low risk og bias** | **Low risk of bias** | **High risk of bias** |
| Shuhaimi, F., et al. (2012) | **Low risk of bias** | **High risk of bias** | **Low risk of bias** | **High risk of bias** | **Low risk of bias** | **Unclear risk of bias** | **Low risk of bias** |
| Aniza, I., et al.(2009) | **Low risk of bias** | **High risk of bias** | **Low risk of bias** | **Low risk of bias** | **Low risk of bias** | **Unclear risk of bias** | **Low risk of bias** |
| Martin, M. A., et al. (2018) | **Low risk of bias** | **High risk of bias** | **High risk of bias** | **High risk of bias** | **Low risk of bias** | **Low risk of bias** | **High risk of bias** |
| Meyer, S. C. (2016) | **Unclear risk of bias** | **High risk of bias** | **High risk of bias** | **Low risk of bias** | **Unclear risk of bias** | **Low risk of bias** | **High risk of bias** |
| Nie, P., et al.(2014). | **Low risk of bias** | **High risk of bias** | **High risk of bias** | **High risk of bias** | **Low risk of bias** | **Low risk of bias** | **High risk of bias** |
| Anderson, P. M. (2012) | **Unclear risk of bias** | **Unclear risk of bias** | **High risk of bias** | **Unclear risk of bias** | **High risk of bias** | **Low risk of bias** | **High risk of bias** |
| Taylor, A. W., et al. (2012) | **Low risk of bias** | **High risk of bias** | **High risk of bias** | **High risk of bias** | **Low risk of bias** | **Unclear risk of bias** | **High risk of bias** |
| Koca, T., et al. (2017) | **Low risk of bias** | **High risk of bias** | **Low risk of bias** | **Unclear risk of bias** | **Low risk of bias** | **Unclear risk of bias** | **Low risk of bias** |
| Ben-Shalom, Y. (2010). | **Low risk of bias** | **High risk of bias** | **High risk of bias** | **Low risk of bias** | **High risk of bias** | **Low risk of bias** | **High risk of bias** |
| Gwozdz, W., et al. (2013) | **Unclear risk of bias** | **High risk of bias** | **Low risk of bias** | **High risk of bias** | **Low risk of bias** | **Low risk of bias** | **Low risk of bias** |
| Datar, A., et al. (2014). | **Low risk of bias** | **High risk of bias** | **High risk of bias** | **Low risk of bias** | **Low risk of bias** | **Low risk of bias** | **Low risk of bias** |
| Parker, M. S. (2007) | **Unclear risk of bias** | **High risk of bias** | **Low risk of bias** | **High risk of bias** | **Low risk of bias** | **Unclear risk of bias** | **High risk of bias** |
| Raheeq, W. et al. (2020) | **Low risk of bias** | **High risk of bias** | **High risk of bias** | **Unclear risk of bias** | **Unclear risk of bias** | **High risk of bias** | **High risk of bias** |
| Ferrari et al. (2016) | **Low risk of bias** | **Unclear risk of bias** | **Low risk of bias** | **High risk of bias** | **Low risk of bias** | **High risk of bias** | **High risk of bias** |
| Pearson et al. (2009) | **Low risk of bias** | **Unclear risk of bias** | **Low risk of bias** | **High risk of bias** | **Low risk of bias** | **Low risk of bias** | **Low risk of bias** |
| Wijtzes et al. (2014) | **Low risk of bias** | **High risk of bias** | **High risk of bias** | **Low risk of bias** | **Low risk of bias** | **Low risk of bias** | **Low risk of bias** |
| Lopoo et al. (2007) | **Low risk of bias** | **High risk of bias** | **High risk of bias** | **Low risk of bias** | **Low risk of bias** | **Low risk of bias** | **Low risk of bias** |
| Richards et al. (1994) | **Low risk of bias** | **High risk of bias** | **High risk of bias** | **High risk of bias** | **Low risk of bias** | **Low risk of bias** | **High risk of bias** |

**Summary of Risk of bias score:**

**High risk of bias: 24 (57.14%)**

**Unclear risk of bias: 01 (2.38%)**

**Low risk of bias: 17 (40.47%)**

**Table 2***

|  | **Selection bias** | **Performance bias** | **Detection bias** | **Attrition bias** | **Selective reporting bias** | **Other bias** |
| --- | --- | --- | --- | --- | --- | --- |
| **High risk of bias** | **2 (4.87%)** | **29 (70.73%)** | **21 (51.21%)** | **17 (41.46%)** | **7 (17.07%)** | **6 (14.63%)** |
| **Low risk of bias** | **23 (56.09%)** | **3 (7.31.87%)** | **18(43.90%)** | **10(24.39%)** | **29 (70.73%)** | **26(63.41%)** |
| **Unclear risk of bias** | **16 (39.02%)** | **9 (21.95%)** | **2 (4.87%)** | **14(34.14%)** | **5(12.19%)** | **9 (21.95%)** |

***Total studies =41 since one Qual study is not counted here**

**JBI Critical Appraisal Checklist for Qualitative Research**

Reviewer…………………………………………………….. Date…21/01/2020………………….

Author **:** Park, S., et al. (2014)................... …………………….. Record Number…11403…...

Yes No Unclear Not applicable

1.Is there congruity between the stated philosophical

perspective and the research methodology? Y□ □ □ □

2. Is there congruity between the research methodology

and the research question or objectives? Y□ □ □ □

3. Is there congruity between the research methodology

and the methods used to collect data? Y□ □ □ □

4. Is there congruity between the research methodology

and the representation and analysis of data? Y□ □ □ □

5. Is there congruity between the research methodology

and the interpretation of results? Y□ □ □ □

6. Is there a statement locating the researcher culturally

or theoretically? Y□ □ □ □

7. Is the influence of the researcher on the research, and

vice- versa, addressed? □ □ UC□ □

8. Are participants, and their voices, adequately

represented? Y□ □ □ □

9. Is the research ethical according to current criteria or,

for recent studies, and is there evidence of ethical

approval by an appropriate body? Y□ □ □ □

10. Do the conclusions drawn in the research report flow

from the analysis, or interpretation, of the data? Y□ □ □ □

**Comments:** Low risk of bias

^Reproduced from: JBI (2014a)
