# Supplementary material for: Dietary habits, physical activity, and sedentary behaviour of children of employed mothers: A systematic review
Source: Prev Med Rep. 2021 Oct 22;24:101607. doi: 10.1016/j.pmedr.2021.101607 (PMC8683879; doi:10.1016/j.pmedr.2021.101607)
Supplement: Supplementary data 3 [file mmc3.docx]

**Title: Dietary habits, physical activity, and sedentary behaviour of children of employed mothers: a systematic review**

**Table Cochrane Collaboration’s tool for assessing risk of bias**

| Bias domain | Source of bias | Support for judgement | Review authors’ judgment (assess as low, unclear or high risk of bias) |
| --- | --- | --- | --- |
| Selection bias | Random sequence generation | Describe the method used to generate the allocation sequence in sufficient detail to allow an assessment of whether it should produce comparable groups |  |
|  | Allocation concealment | Describe the method used to conceal the allocation sequence in sufficient detail to determine whether intervention allocations could have been foreseen before or during enrolment |  |
| Performance bias | Blinding of participants and personnel* | Describe all measures used, if any, to blind trial participants and researchers from knowledge of which intervention a participant received. Provide any information relating to whether the intended blinding was effective |  |
| Detection bias | Blinding of outcome assessment* | Describe all measures used, if any, to blind outcome assessment from knowledge of which intervention a participant received. Provide any information relating to whether the intended blinding was effective |  |
| Attrition bias | Incomplete outcome data* | Describe the completeness of outcome data for each main outcome, including attrition and exclusions from the analysis. State whether attrition and exclusions were reported, the numbers in each intervention group (compared with total randomised participants), reasons for attrition or exclusions where reported, and any reinclusions in analyses for the review |  |
| Reporting bias | Selective reporting |  |  |
| Other bias | Anything else, ideally prespecified | State any important concerns about bias not covered in the other domains in the tool |  |

*Assessments should be made for each main outcome or class of outcomes.

Ref: Higgins, J., Altman, D., Gotzsche, P., Juni, P., Moher, D., & Oxman, A. et al. (2011). The Cochrane Collaboration's tool for assessing risk of bias in randomised trials. *BMJ*, *343*(oct18 2). https://doi.org/10.1136/bmj.d5928
