# Supplementary material for: Dietary habits, physical activity, and sedentary behaviour of children of employed mothers: A systematic review
Source: Prev Med Rep. 2021 Oct 22;24:101607. doi: 10.1016/j.pmedr.2021.101607 (PMC8683879; doi:10.1016/j.pmedr.2021.101607)
Supplement: Supplementary data 4 [file mmc4.docx]

**Title: Dietary habits, physical activity, and sedentary behaviour of children of employed mothers: a systematic review**

**JBI Critical Appraisal Checklist for Qualitative Research**

Reviewer……………………………………………………. Date…………………….

Author …………………….. Record Number…...

Yes No Unclear Not applicable

1.Is there congruity between the stated philosophical

perspective and the research methodology?

2. Is there congruity between the research methodology

and the research question or objectives?

3. Is there congruity between the research methodology

and the methods used to collect data?

4. Is there congruity between the research methodology

and the representation and analysis of data?

5. Is there congruity between the research methodology

and the interpretation of results?

6. Is there a statement locating the researcher culturally

or theoretically?

7. Is the influence of the researcher on the research, and

vice- versa, addressed?

8. Are participants, and their voices, adequately

represented?

9. Is the research ethical according to current criteria or,

for recent studies, and is there evidence of ethical

approval by an appropriate body?

10. Do the conclusions drawn in the research report flow

from the analysis, or interpretation, of the data?

**Comments:** Low risk of bias

^Reproduced from: JBI (2014a)
